# Does Self-Sacrifice Make Me Great? Research on the Relationship Between Employee Conscientiousness and Pro-Social Rule Breaking

**DOI:** 10.3389/fpsyg.2022.834274

**Published:** 2022-05-30

**Authors:** Xiayi Liu, Hongqing Wang, Xiajun Liu

**Affiliations:** ^1^School of Business Administration, Huaqiao University, Quanzhou, China; ^2^School of Business, Nanjing Audit University, Nanjing, China

**Keywords:** duty orientation, achievement orientation, leader reward omission, job autonomy, pro-social rule breaking

## Abstract

Based on the theory of purposeful work behavior, this study proposed that the two facets of employee conscientiousness, namely duty orientation and achievement orientation, have opposite effects on pro-social rule breaking (PSRB). We also explored the moderating effect of employees’ task characteristic (job autonomy) and social characteristic (leader reward omission) on the above relationships. Using two-wave data collected from 216 employee-supervisor dyads, we found that duty orientation was positively related to PSRB, while achievement orientation was negatively related to PSRB. Further, job autonomy, by amplifying employees’ perceived meaningfulness of their higher-order implicit goals, can strengthen the positive effect of duty orientation and the negative effect of achievement orientation on PSRB. Similarly, leader reward omission could also activate the negative effect of achievement orientation and PSRB, but not significantly moderate the positive relationship between duty orientation and PSRB. By separating the distinct role of facet-specific personality, our study sheds light on the relationship between employee conscientiousness and PSRB.

## Introduction

Conscientiousness has previously been observed to have a negative impact on destructive employees’ workplace deviance (Kluemper et al., 2015; Guay et al., 2016). This is not surprising considering that conscientious individuals are self-disciplined, careful, and morally scrupulous (Barrick and Mount, 1991; Costa et al., 1991). In line with this view, current researchers reasoned consistently that conscientious employees are less likely to engage in pro-social rule breaking (PSRB) behaviors (Dahling et al., 2012; Vardaman et al., 2014).

Yet, our study argues that we should take a deeper exploration and research on the relationship between employee conscientiousness and PSRB for the following two reasons: Firstly, there are significant differences between workplace deviance and PSRB in their intrinsic motivation. Workplace deviance is mainly driven by employees’ self-interested or hostile motives (Judge et al., 2006), whereas PSRB “is characterized by voluntary divergence from organizational norms with honorable intentions to benefit the organization or its stakeholders.” (Dahling et al., 2012, p. 22). Therefore, PSRB is prone to induce a mixed motivational situation for individual employee: he/she must select between violating organizational rules and regulations to help their organization or turning a blind eye to avoid the risk of punishment or social ostracization associated with PSRB (Vardaman et al., 2014). Secondly, conscientiousness proved to comprise more narrow dimensions, which mark important individual differences (e.g., Costa et al., 1991; Moon, 2001; Roberts et al., 2005). Researchers ignoring those differences might not achieve maximum validity when examining the effects of conscientiousness on work-related outcomes, especially workplace behaviors involving a decision dilemma (Ashton, 1998; Moon, 2001; Chae et al., 2019). Hence, taking conscientiousness as a general factor might insufficiently reveal its effect on PSRB.

We try to address this issue by further exploring the relationship between conscientiousness and PSRB by adopting the narrow-trait approach. Specifically, due to the motivational dilemma involving PSRB, we divide conscientiousness into two well-established distinct facets, namely duty orientation (other-centered) and achievement orientation (self-centered) (Moon, 2001). Differentiating these two facets is critical given their disparate implications for similar interpersonal dilemmatic situations, such as knowledge sharing (Chae et al., 2019), voice behavior (Tangirala et al., 2013), commitment escalation (Moon, 2001), et al. Basing on the theory of purposeful work behavior (TPWB) (Barrick et al., 2013), we propose that duty orientation is positively related to PSRB due to its other-centered implicit high-order goals, whereas achievement orientation is negatively related to PSRB for its self-centered purposeful motivational strivings.

Further, according to TPWB, work environments can facilitate or hind the effect of personality traits on individuals’ purposeful work striving. Generally, these environments can be broken down into two main categories: task characteristic and social characteristic (Barrick et al., 2013). As one of the five core components of work characteristics, job autonomy gives employees freedom in carrying out work. Employees with higher job autonomy should have a greater influence on how a task is performed (Hackman and Oldham, 1975). In this regard, they are more likely to choose behaviors following their implicit high-order goals. Besides, employees also make judgments about their behaviors according to information sources provided by their leaders (He et al., 2020a). Leader reward omission, a common passive–avoidant type of leadership behavior (Peng et al., 2021; Wang et al., 2021), should serve as a critical social characteristic that influences employees’ perceived risks and costs associated with PSRB. Therefore, this study chooses job autonomy as a task characteristic and leader reward omission as a social characteristic to further verify that the two facets of conscientiousness have opposite effects on PSRB.

By doing so, our research makes two major theoretical contributions to existing literature. Firstly, we extend understanding regarding the relationship between employee conscientiousness and PSRB. Prior studies found that conscientiousness, as a general factor, is negatively related to PSRB (Dahling et al., 2012; Vardaman et al., 2014). However, we found that the two narrow traits of conscientiousness, namely duty orientation and achievement orientation, have disparate effects on PSRB. By delineating the distinct roles of the two facets of conscientiousness, our study helps people to better understand conscientious employee’s choice under dilemmatic situations, echoing prior calls for a narrow use of personality in the workplace (Ashton, 1998; Moon, 2001; Dudley et al., 2006). Secondly, we provide initial evidence regarding the validity of TPWB (Barrick et al., 2013). Our results support the notion that the high-order implicit goals associated with traits will guide and direct the unique patterns of people’s thoughts, emotions, and behavior. Moreover, our study further validates TPWB by examining the moderating role of job autonomy as a task characteristic and leader reward omission as a social characteristic.

## Theory and Hypotheses

### Theory of Purposeful Work Behavior

To explicate the disparate effects, this study adopts TPWB (Barrick et al., 2013) as the overarching theoretical framework. A core tenet of TPWB is that it is the implicit high-order goals associated with the five-factor-model (FFM) traits that determine an individual’s experienced meaningfulness, which, in turn, triggers motivated workplace behavior. In addition, TPWB posits that an individual’s task and social characteristics may facilitate his or her perceived meaningfulness when they act in concert with the purposeful work strivings (Barrick et al., 2013). Briefly, TPWB proposed that an employee’s workplace behavior depends on the joint effects of their personality traits and job characteristics. Prior studies have adopted this theory to explain personality differences in predicting various workplace outcomes, such as work-email activity, job performance, work engagement, organizational citizenship behaviors, and counterproductive workplace behaviors (Frieder et al., 2018; Smith and Denunzio, 2019; Er and Saw, 2020). Taking into account the differential motivations underlying duty orientation (other-centered) and achievement orientation (self-centered), we believe TPWB is appropriate for revealing the distinct effects of duty orientation and achievement orientation on PSRB as well as the moderating role of job autonomy and leader reward omission.

### Two Facets of Conscientiousness and PSRB

PSRB refers to those behaviors that employees actively violate formal organization regulations, policies, or prohibitions with the aim to promote the well-being of the whole organization or thier stakeholders (Morrison, 2006). Employees often fall into a moral dilemma when facing the decision-making situation involving PSRB. Employees who choose to engage in PSRB might help improve customer satisfaction, ameliorate organization structure and management system, and promote work quality, while they themselves might be punished for violating organizational rules and regulations or receive unfavorable evaluations from their leaders and colleagues (Morrison, 2006; Dahling et al., 2012). For those who choose to avoid PSRB, they can protect their own interests at the expense of the organization’s or stakeholders’ benefits (Morrison, 2006; Vardaman et al., 2014). Taking together, due to the dilemmatic nature of PSRB, whether or not engaging in this behavior is largely determined by one’s inner pursuit.

According to TPWB, we first argue that duty orientation is positively related to PSRB. Although employees with duty orientation and achievement orientation are all characterized by diligence and having high performance in their work, the implicit high-order goals behind them are of significant differences. Employees with higher duty orientation tend to be other-centered, adhere to ethical principles, and persist in doing what they believe is right. Prior studies found that those employees are more likely to avoid escalation of commitment at the expense of their personal reputation (Moon, 2001), to voice even if it might elicit their leaders’ antipathy and threaten their self-image (Tangirala et al., 2013), and to engage in knowledge-sharing behavior, despite the threat of personal value or privilege loss (Chae et al., 2019). According to TPWB, we suggest that the other-centered motivation behind duty-oriented employees is more likely to prompt them to engage in PSRB behavior. This is because such behavior is beneficial for the organization’s and its stakeholders’ benefits (Morrison, 2006), which is in accord with their implicit high goals. As such, we proposed that:

H1: Duty orientation is positively related to PSRB behavior.

By contrast, we argue that achievement-oriented employees are more likely to avoid PSRB. Employees with higher achievement orientation are self-centered and care more about personal career success. Although they are also very hardworking and efficient at work, they are more concerned about personal gains and losses associated with their behavior and often evaluate whether such behavior is conducive to leadership emergence (Marinova et al., 2013). Consequently, such employees are reluctant to engage in behaviors that are beneficial for others while risky to themselves. For instance, they will remain silent because of the personal risks caused by voice behavior (Tangirala et al., 2013), hide their knowledge for the potential position or privilege loss risk after knowledge-sharing (Chae et al., 2019; He P. et al., 2021), or even turn a blind eye to the difficulties encountered by colleagues with the aim to outperform others (Marinova et al., 2013). According to TPWB, when facing the PSRB dilemma, achievement-oriented employees are more likely to avoid this behavior because such behavior is risky to themselves, which is inconsistent with their implicit self-center goals. As such, we proposed that:

H2: Achievement orientation is negatively related to PSRB behavior.

### Moderating Effects of Job Autonomy and Leader Reward Omission

Personality literature has long suggested that research on the effects of individual personality should not ignore the role of environmental factors (Funder, 2006; Zaccaro et al., 2018). TPWB holds that external environmental factors can activate or inhibit individuals’ sense of the meaningfulness of the goals they pursued and which, in turn, affect their work behavior (Barrick et al., 2013). As stated above, job autonomy and leader reward omission represent critical task and social characteristics, respectively, that influence employees’ perceived meaningfulness of their implicit high-order goals. Hence, this paper examines the moderating roles of job autonomy and leader reward omission to further verify H1 and H2.

Job autonomy refers to the degree to which employees perceive themselves to be able to control and determine working methods, arrangements, and standards (Breaugh, 1985). When employees perceive higher levels of job autonomy in their work, they feel less control from their leaders or organizations, and more freedom to determine what to do in their daily work (Morgeson et al., 2005). Given the voluntary, while rule-violating nature of PSRB, we proposed that employees with high job autonomy are more likely to make decisions about engaging in PSRB or not in accordance with their implicit higher-order goals. Unlike compulsory citizenship behavior (He et al., 2020b), the voluntary nature of PSRB leaves some space for employees to decide to engage in it or not.

In this case, higher job autonomy might amplify the impact of personal motivation on workplace behaviors (Mischel, 1977; Barrick et al., 2013). Specifically, for duty-oriented employees, higher job autonomy allows them to insist on doing what they believe to be right. As such, when facing a PSRB dilemma, they are more likely to engage in such behavior because it can satisfy their implicit other-centered goal. On the contrary, achievement-oriented employees are more likely to avoid PSRB because such behavior is inconsistent with their pursuit of personal success.

On the contrary, when employees perceive lower levels of job autonomy, they feel more control over their work and less freedom for decision-making, which may decrease their sense of organizational responsibility and psychological ownership (Pattnaik and Sahoo, 2021). In this situation, all employees have to do their work according to the organization job description strictly. That is to say, neither duty-oriented employees nor achievement-oriented employees can decide to engage in PSRB or not when facing such a moral dilemma. Hence, we proposed that lower job autonomy might hind the impacts of duty-oriented and achievement-oriented employees’ high-implicit goals on PSRB behavior. Taking together, we argue that high job autonomy could amplify the positive relationship between duty orientation and PSRB and the negative relationship between achievement orientation and PSRB. Based on the above analysis, this article proposed that:

H3a: The positive relationship between duty orientation and PSRB becomes stronger when job autonomy is high.

H3b: The negative relationship between achievement orientation and PSRB becomes stronger when job autonomy is high.

Leader reward omission, a typical passive leadership style, refers to such kind of leaders who do not reward subordinates for their high performance at work (Hinkin and Schriesheim, 2008). In this case, employees might feel that they do not get the praise, encouragement, and respect they deserve, which will consequently be tied to a series of negative results. Prior studies found that leader reward omission can undermine subordinates’ trust toward their leaders, increase employees’ perceived cost at work, and consequently reduce workplace feedback-seeking behavior (Zhang et al., 2020). That is to say, leader reward omission discourages employees’ willingness to take risks and the consequences under uncertain situations (Dirks, 2000). According to TPWB, we suggest that leader reward omission will not affect the relationship between duty orientation and PSRB. The reason is that the other-centered motivation behind duty-orientation employees enables them to be immune to extrinsic rewards and punishments, sticking to doing what they believe to be right. In brief, leader reward omission will not affect duty-oriented employees’ perceived meaningfulness of PSRB. However, leader reward omission will strengthen the negative relationship between employees’ achievement orientation and PSRB. This is because leader reward omission can strengthen achievement-oriented employees’ perceived risks and costs for engaging in PSRB behavior, which runs counter to their implicit high-order goals for personal success. To be specific, high leader reward omission leaves subordinates unable to obtain performance feedback and organizational recognition, this may signal to employees that the organization denies its value. This will make employees feel great threat, insecurity, and inequitable treatment when engaged in PSRB behavior (Wang et al., 2021). Therefore, when leader reward omission is higher, achievement-oriented employees are more likely to avoid engaging in such behavior as much as possible for the sake of promotion safety. Based on the above analysis, this article proposed that:

H4a: Leader reward omission does not moderate the relationship between duty orientation and PSRB.

H4b: The negative relationship between achievement orientation and PSRB becomes stronger when leader reward omission is high.

**FIGURE 1:**
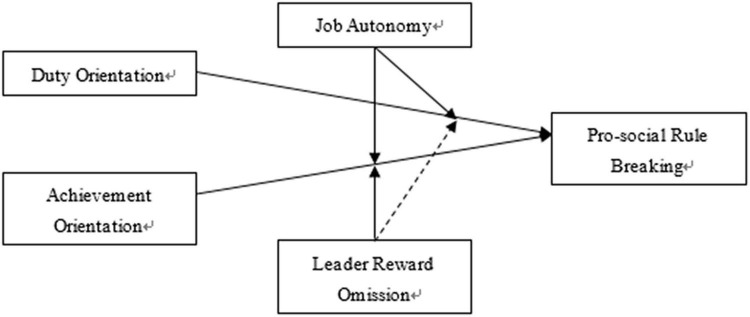
Conceptual model.

To sum up, the conceptual model is displayed in Figure 1.

## Materials and Methods

### Participants and Procedure

To test our hypotheses, we collected data from 4 Chinese companies that represent diverse industries, including manufacturing, insurance, e-commerce, and software. We send out two-stage surveys: a focal employee survey and a supervisor survey. Focal employees rated their demographic characteristics, duty orientation, achievement orientation, leader reward omission, and job autonomy, and three months later, supervisors completed items related to the focal employee’s PSRB. With the assistance of internal coordinators (human resource personnel), we first made clear to participants the scientific research purpose only and the confidentiality of our survey. After completion, participants were instructed to return the survey directly to the researchers, in closed envelopes.

We sent out surveys to 248 employees and their immediate supervisors. 225 employees returned completed surveys, representing a response rate of 90.73%. A total of 231 supervisors’ surveys were received, yielding a response rate of 93.15%. Because of missing data, the final matched sample of employee-supervisor dyads was 216.

Of the 216 employees, 95 (44.0%) were male and 121(56.0%) were female. Age was coded into four categories (along with the percentage of sample in each category): below 30 years (32.4%), 31 to 40 years (26.9%), 41 to 50 years (26.9%), over 51 years (13.9%). In terms of education, 26.9% had a high school diploma or lower, 25.0% had completed a college degree, 33.3% held a bachelor degree, 18.5% had postgraduate qualifications or higher. Tenure was reported for four bands: less than 3 years (24.1%), 4 to 6 years (24.1%), 7 to 9 years (33.3%), over 10 years (18.5%).

### Measures

We used a response format of 5-point scales (1 = strongly disagree to 5 = strongly agree, unless otherwise noted). To ensure all items can be clearly understood by every participant, we translate the English scales into Chinese following a back-translation procedure.

Duty orientation was measured with an established 8-item scale from the 240-item Revised NEO Personality Inventory (Costa and McCrae, 1992). A sample item reads “I adhere strictly to my ethical principles.” The coefficient α in this study was 0.903.

Achievement orientation was also evaluated using 8 items from the 240-item Revised NEO Personality Inventory (Costa and McCrae, 1992). A sample item was “I strive to achieve all I can.” The coefficient α in this study was 0.903.

Job autonomy was rated using 3 items from Spreitzer (1995). A sample item reads “I have significant autonomy in determining how I do my job.” The coefficient α in this study was 0.758.

Leader reward omission was measured using a 6-item scale from Hinkin and Schriesheim (2008). Sample items include “I often perform well in my job and still receive no praise from my manager” and “My good performance often goes unacknowledged by my manager.” The coefficient α in this study was 0.797.

PSRB was measured with a 13-items scale that was developed by Dahling et al. (2012). A sample item reads “This employee breaks organizational rules or policies to do his/her job more efficiently.” The coefficient α in this study was 0.964.

Control variables: To exclude alternative explanations, consistent with previous studies, we selected some demographic variables as the control variables (Bernerth and Aguinis, 2016). Specifically, employee gender (1 = male, 2 = female), age (1 = below 30 years, 2 = 31 to 40 years, 3 = 41 to 50 years, 4 = over 51 years), education (1 = high school diploma or lower, 2 = college degree, 3 = bachelor degree, 4 = postgraduate qualifications or higher), and tenure (1 = less than 3 years, 2 = 4 to 6 years, 3 = 7 to 9 years, 4 = over 10 years).

### Data Analysis

In this study, SPSS statistical software was used for descriptive statistics, reliabilities, and correlations analyses, as well as common method variance, normal distribution, and hypothesis test. Confirmatory factor analyses were performed by Mplus Version 7.4.

## Results

### Confirmatory Factor Analyses

Before testing the proposed model in our study, we first carried out confirmatory factor analyses (CFA) to assess whether the scales used in our study, namely duty orientation, achievement orientation, leader reward omission, job autonomy, and PSRB, have eligible discriminant validity. Based on the methods recommended by Anderson and Gerbing (1988), we firstly tested the Chi-square differences between our five-factor baseline model and five alternative models to see which model mostly fit the data. The results in Table 1 suggested that our five-factor model provided significantly better fit than the other five alternative models (χ^2^ = 1146.998, df = 655, CFI = 0.908, TLI = 0.901, RMSEA = 0.059) (Steiger, 1990; Kline, 2015). As such, the results support the distinctiveness of key variables, enhancing our confidence in testing the following hypotheses.

**TABLE 1:** Results of the confirmatory factor analyses of study variables.

Model	χ^2^	*df*	χ^2^/*df*	*CFI*	*TLI*	*RMSEA*	Δ χ^2^(Δ *df*)
5-factor:DO, AO, RO, JA, PSRB	1146.998	655	1.75	0.908	0.901	0.059	-
4-factor:DO, AO, RO + JA, PSRB	1360.644	659	2.06	0.869	0.860	0.070	213.646 (4)
4-factor:DO + AO, RO, JA, PSRB	1936.315	659	2.94	0.761	0.745	0.095	789.317 (4)
3-factor:DO + AO + PSRB, RO, JA	2852.993	662	4.32	0.590	0.564	0.124	1705.995 (7)
2-factor:DO + AO + PSRB, RO + JA	3056.580	664	4.60	0.552	0.526	0.129	1909.582 (9)
1-factor:DO + AO + PSRB + RO + JA	3365.627	665	5.06	0.494	0.465	0.137	2218.629 (10)

*n = 216. DO, duty orientation; AO, achievement orientation; RO, leader reward omission; JA, job autonomy.*

### Descriptive Statistics

Table 2 provided the descriptive statistics, reliabilities, and correlations of the variables in our study. Consistent with our hypotheses, duty orientation was positively related to PSRB (*r* = 0.162, *p* < 0.05), whereas achievement orientation had a negative correlation with PSRB (*r* = −0.237, *p* < 0.001), which provides preliminary support for H1 and H2. The test of normal distribution mainly includes two elements, namely skewness and kurtosis. Those with skewness value less than 3 and kurtosis value less than 10 can be considered as basically conforming to a normal distribution (Kline, 2015). In this study, the maximum skewness of each variable is 2.443, and the maximum kurtosis is 5.277. The results suggest that our data conform to normal distribution.

**TABLE 2:** Descriptive statistics and correlations for study variables.

Variables	*M*	*SD*	1	2	3	4	5	6	7	8
(1). Gender *^a^*	0.44	0.50								
(2). Age	2.22	1.05	0.079							
(3). Education	2.28	0.94	0.071	0.167*						
(4). Tenure	2.46	1.05	–0.071	0.058	0.088					
(5). DO	3.60	0.76	–0.075	−0.153*	–0.022	–0.041				
(6). AO	3.55	0.77	−0.213**	−0.183**	−0.161*	–0.110	0.256***			
(7). RO	2.81	0.54	0.077	–0.029	0.084	0.046	–0.018	–0.063		
(8). JA	2.77	0.98	–0.132	−0.221**	0.103	0.033	0.085	0.076	–0.092	
(9). PSRB	1.66	0.74	0.096	0.027	0.429***	0.177**	0.162*	−0.237***	0.004	0.298***

n = 216.*p < 0.05, **p < 0.01, ***p < 0.001(two-tailed). ^a^ Employee gender was coded as 0, female; 1, male.

### Common Method Variance

In order to test for common method bias, we performed Harman’s single-factor test. The unrotated factor analysis results revealed that the first factor only accounts for 26.41% of the variance, which is well below the threshold of 50%. Thus, common method variance is not a serious threat in our study.

### Hypothesis Testing

We first performed a multicollinearity test before hypotheses testing. The results showed that variance inflation factor (VIF) values of each model were between 1.030 and 1.172, and tolerance coefficients were between 0.880 and 0.971, indicating that there was no serious multicollinearity problem in our study. We formally tested our hypothesized model using hierarchical regression analysis (see Table 3). The results of M2 in Table 3 suggested that after controlling for employees’ gender, age, education, and tenure, duty orientation was positively related to PSRB (β = 0.227, p < 0.001) while achievement orientation was negatively related to PSRB (β = −0.216, p < 0.01). This supported our hypotheses that the two facets of conscientiousness had opposite effects on PSRB. Thus, H1 and H2 were confirmed.

**TABLE 3:** Results of hierarchical regression modeling equation predicting PSRB.

	PSRB									

Variable	M_1_	M_2_	M_3_		M_4_		M_5_		M_6_	
						
			M_3(a)_	M_3(b)_	M_4(a)_	M_4(b)_	M_5(a)_	M_5(b)_	M_6(a)_	M_6(b)_
Gender	0.081	0.053	0.124*	0.113	0.080	0.067	0.097	0.089	0.052	0.038
Age	−0.058	−0.055	0.029	0.030	−0.017	−0.014	−0.034	−0.040	−0.084	−0.087
Education	0.420***	0.393***	0.379***	0.379***	0.359***	0.326***	0.422***	0.423***	0.406***	0.406***
Tenure	0.149*	0.135*	0.149*	0.136*	0.124*	0.118*	0.158*	0.141*	0.134*	0.129*
DO		0.227***	0.168**	0.173**			0.179**	0.172**		
AO		−0.216**			−0.173**	−0.197**			−0.165***	−0.178**
JA			0.263***	0.255***	0.277***	0.260***				
RO							−0.043	−0.027	−0.053	−0.022
DO*JA				0.165**						
AO*JA						−0.142*				
DO*RO								−0.106		
AO*RO										-0.204**
*R* ^2^	0.213	0.284	0.307	0.334	0.307	0.325	0.246	0.257	0.239	0.280
Δ*R*^2^		0.071	0.094	0.027	0.094	0.018	0.033	0.011	0.026	0.041
*F*	14.257***	13.819***	15.441***	14.892***	15.441***	14.295***	11.363***	10.256***	10.960***	11.538***

n = 216. *p<0.05, **p<0.01, ***p<0.001 (two-tailed).

**FIGURE 2:**
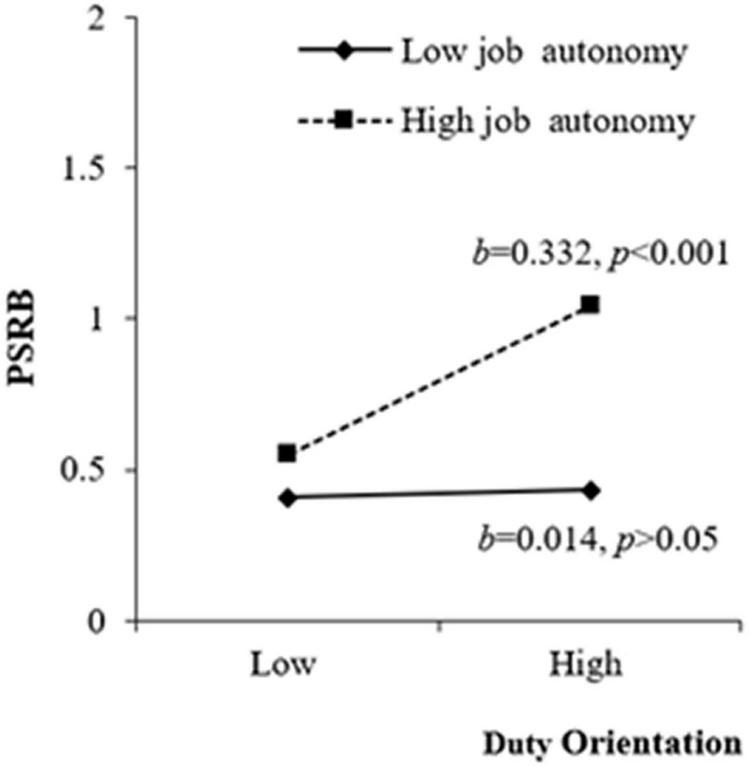
Moderating effect of job autonomy between duty orientation and PSRB.

**FIGURE 3:**
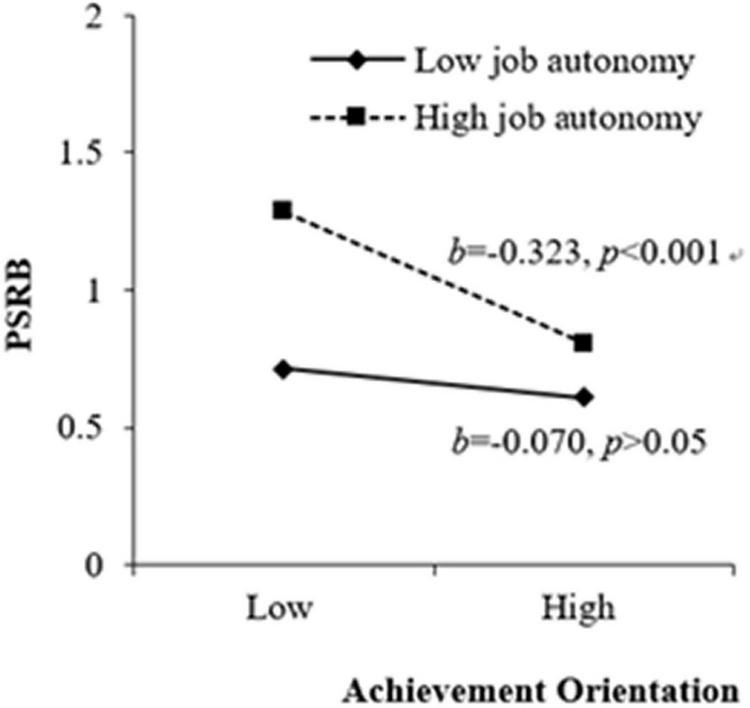
Moderating effect of job autonomy between achievement orientation and PSRB.

H3a and H3b stated that job autonomy strengthened the positive and negative effects of duty orientation and achievement orientation on PSRB, respectively. The results of M3(b) in Table 3 indicated that job autonomy positively and significantly moderated the positive relationship between duty orientation and PSRB (β = 0.165, *p* < 0.01), and the results of M4(b) in Table 3 revealed that job autonomy negatively and significantly moderated the negative relationship between achievement orientation and PSRB (β = −0.142, *p* < 0.05). We plotted the interactions to better illustrate the moderation effects (as shown in Figures 2, 3; Aiken and West, 1991). In Figure 2, simple slope analyses suggested that there was a positive relationship between duty orientation and PSRB when job autonomy was high (1 *SD* above the mean; β = 0.332, *t* = 4.105, *p* < 0.001) but a non-significant relationship when job autonomy was low (1 *SD* below the mean; β = 0.014, *t* = 0.184, *p* > 0.05). As such, H3a was supported. In Figure 3, simple slope analyses revealed that achievement orientation and PSRB was negatively related when job autonomy was high (1 *SD* above the mean; β = −0.323, *t* = −3.689, *p* < 0.001) but was insignificantly related when job autonomy was low (1 *SD* below the mean; β = −0.070, *t* = −0.946, *p* > 0.05), supporting H3b.

**FIGURE 4:**
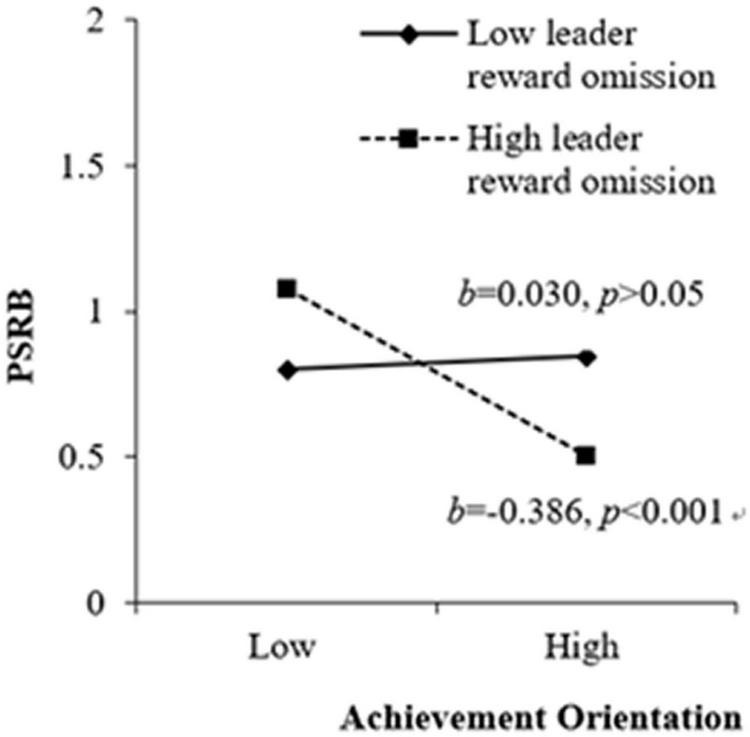
Moderating effect of leader reward omission between achievement orientation and PSRB.

H4a and H4b posited that leader reward omission did not moderate the positive relationship between duty orientation and PSRB while strengthened the negative effects of achievement orientation on PSRB, respectively. As presented in M5(b) in Table 3, leader reward omission had no moderating effect on the relationship between duty orientation and PSRB (β = −0.106, *p* > 0.05), yet a negative moderating effect on the relationship between achievement orientation and PSRB (β = −0.204, *p* < 0.01). Figure 4 shows that achievement orientation was negatively related to PSRB only when leader reward omission was high (1 *SD* above the mean; β = −0.386, *t* = −4.303, *p* < 0.001), but this relationship was insignificant when leader reward omission was low (1 *SD* below the mean; β = 0.030, *t* = 0.358, *p* > 0.05). Thus, hypothesis 4a and hypothesis 4b received support.

## Discussion

Using TPWB as an overarching framework, the present study examines the effects of two different facets of employees’ conscientiousness (duty orientation and achievement orientation) on a common workplace dilemma, namely, PSRB. Further, we also adopt job autonomy and leader reward omission as important boundary conditions to verify the above relationships. Our empirical results suggested that: (1) Duty orientation positively predicts PSRB, while achievement orientation negatively predicts PSRB. (2) Job autonomy positively moderates the relationship between duty orientation and PSRB, and negatively moderates the relationship between achievement orientation and PSRB. (3) Leader reward omission negatively moderates the relationship between achievement orientation and PSRB, but does not moderate the relationship between duty orientation and PSRB significantly. Our results expand and enrich previous research results about the linkage between employees’ conscientiousness and PSRB behavior (Morrison, 2006; Vardaman et al., 2014), which has strong theoretical and practical implications for theory development and management practice.

### Theoretical Implications

This study contributes to existing research in three major ways. Firstly, our findings help clarify the relationship between employee conscientiousness and PSRB behavior by highlighting the disparate effects of duty orientation and achievement orientation. Prior studies have demonstrated a consistent relationship between a broad factor of conscientiousness and PSRB (Morrison, 2006; Vardaman et al., 2014). While this might conceal the divergent impact of different facets of conscientiousness on PSRB. As suggested, “a more narrow use of conscientiousness will be beneficial” (Moon, 2001, p. 537) when examining its effects on workplace behaviors that involves self-orientation and other-orientation. Accordingly, our study addresses the repeated calls for research to analyze the effects of personality traits on a narrower conceptualization (Hogan and Roberts, 2015).

Secondly, our study provides a deeper and more comprehensive understanding on the intention of PSRB. Most empirical studies view PSRB as a kind of risky behavior that violates organizational rules and regulations (Wang and Shi, 2021), which is not consistent with Morrison’s (2006) original definition, where PSRB was regarded as a social dilemma for agents themselves. Drawing on TPWB, our study empirically reveals that PSRB is actually a moral dilemma for conscientious employees from the implicit higher-order goals view. In this regard, our study helps researchers to better understand the nature of PSRB.

Thirdly, our study also contributes to the literature on situational variation in trait expression. Prior studies have stressed the critical role of situations in conditioning the impact of traits on behaviors (Funder, 2006; Zaccaro et al., 2018). Our study addresses this call by examining the moderating of job autonomy and leader reward omission. Specifically, we found that job autonomy might amplify the positive effect of duty orientation and the negative of achievement orientation on PSRB, and leader reward omission could strengthen the negative of achievement orientation on PSRB. These results demonstrate the necessity and importance of incorporating task and social characteristics as contextual factors in examining the effects of certain personality traits.

### Practical Implications

Our study also yields several important practical implications. Firstly, managers should pay attention to discern various facets of conscientiousness, which might lead to divergent outcomes. Although most studies have consistently stressed the critical role of conscientiousness in improving employees’ work performance, our results suggest that such a view might be misleading. As shown in our study, conscientious employees with duty orientation are more likely to engage in PSRB to promote the benefits of the organization and stakeholders, despite the personally risky potential associated with such behavior. On the contrary, conscientious employees with achievement orientation tend to avoid PSRB for the sake of their own interests. That is to say, employees might be conscientious for different intentions. As such, organizations that require employees’ PSRB in the turbulent situation must learn how to evaluate the various motivations behind employees’ conscientiousness.

Secondly, our study also suggests that job autonomy and leader reward omission can serve as critical boundary conditions on the relationship between the two facets of conscientiousness and PSRB. In this case, supervisors should delegate more authority to duty-orientated employees to stimulate their PSRB. By contrast, to promote achievement-orientated employees’ PSRB behavior, supervisors should pay more attention to monitoring their contribution to the job and appreciate their good performance on time. These practices can help prevent achievement-orientated employees from being overly self-interested and turning a blind eye to the benefits of the whole organization.

Thirdly, organizations should be more tolerant toward employees’ PSRB behavior. Due to the moral dilemmatic nature of PSRB, it is not hard to conclude that employees who choose to engage in PSRB put organizational interests ahead of their personal benefits. In this case, these employees should not be punished severely for violating organizational rules and regulations. On the contrary, organizations should show respect to and reward employees for engaging in PSRB bravely. If so, other employees can also choose to engage in PSRB when facing a similar moral dilemma, which can significantly improve organizational flexibility and competitiveness.

### Limitations and Future Research Directions

There are still some limitations of this study that should be noted. Firstly, although we adopted a two-stage, multi-source method to collect data, the duty orientation, achievement data, job autonomy, and leader reward omission data were all collected from employees. As such, there is inevitably common method bias and socially desirable responding in our data. Hence, future research can use experimental methods, objective data, or archival data to repeat this research to improve the robustness of our results. Secondly, we did not incorporate binary tenure as a control variable in our study. Binary tenure refers to the period in which a follower had worked with his or her leader. Although some prior studies have shown that dyadic tenure has no significant effect on PSRB (Zhu et al., 2018; Chen et al., 2019; He B. et al., 2021), we believe binary tenure might have a great impact on leader-member exchange and guanxi, which, in turn, influence employees’ psychological safety in engaging extra-role workplace behaviors (Sparrowe and Liden, 2005; He et al., 2019). As such, we call for future research to control binary tenure in their studies regarding employee-supervisor dyads. Thirdly, our research paid little attention to the intervening mechanisms between the two facets of conscientiousness and PRSB. Actually, duty orientation and achievement orientation might influence employees’ PSRB through different paths. For example, duty orientation and achievement orientation may influence PSRB through different role identification (Tangirala et al., 2013). Finally, future studies can explore the relationship between employee conscientiousness and PSRB by identifying critical boundary conditions. By revealing the disparate effects of the two facets of conscientiousness on PSRB, this paper provides a deeper understanding of the relationship. However, future research can further explore whether employee conscientiousness has opposite effects on PSRB under different levels of the same boundary condition, such as environmental uncertainty.

## Data Availability Statement

The raw data supporting the conclusions of this article will be made available by the authors, without undue reservation.

## Ethics Statement

Written informed consent was obtained from the individual(s) for the publication of any potentially identifiable images or data included in this article.

## Author Contributions

XyL wrote the original draft of the manuscript and analyzed the data. HW and XjL revised the manuscript. All authors contributed to the design and conceptualization of the manuscript, as well as to reviewing and editing the manuscript.

## Conflict of Interest

The authors declare that the research was conducted in the absence of any commercial or financial relationships that could be construed as a potential conflict of interest.

## Publisher’s Note

All claims expressed in this article are solely those of the authors and do not necessarily represent those of their affiliated organizations, or those of the publisher, the editors and the reviewers. Any product that may be evaluated in this article, or claim that may be made by its manufacturer, is not guaranteed or endorsed by the publisher.
